# Effect of *1Bx7null* on Soft Wheat Cookie Quality Under Different Nitrogen Inputs and Its CAPS Marker Development

**DOI:** 10.3390/foods14234137

**Published:** 2025-12-02

**Authors:** Pingping Zhang, Guicheng Song, Yuntao Zhang, Jinbao Yao

**Affiliations:** 1 Institute of Food Crops, Jiangsu Academy of Agricultural Sciences, Nanjing 210014, China; 2 Collaborative Innovation Center for Modern Crop Production Co-Sponsored by Province and Ministry, Nanjing 210014, China; 3 Institute of Agricultural Science and Technology, Jiangsu Hongze Lake Farm Group Co., Ltd., Suqian 223900, China

**Keywords:** soft wheat, *1Bx7null*, nitrogen, end-use quality, cookie quality, CAPS marker

## Abstract

The role of HMW-GSs in soft wheat quality remains inadequately understood. In Chinese soft wheat variety Ningmai 9, a nonsense mutation (*Glu-B1x*, *1Bx7null*) reduced dough tenacity while enhancing dough extensibility under both low (LN) and high nitrogen (HN) inputs. The improved extensibility in the NIL carrying *1Bx7null* was primarily due to a reduced glutenin/gliadin ratio, with HN further increasing extensibility compared to LN. Notably, the NIL under HN exhibited better cookie quality than WT under LN without yield loss (*p* < 0.05). A CAPS marker was developed based on a C-to-T SNP at 514 bp in the CDS of *1Bx7null*, reliably distinguishing *1Bx7* and *1Bx7null* alleles. This allele-marker combination shows promising potential for soft wheat breeding. Future studies should explore the effects of allele *1Bx7null* across diverse genetic backgrounds and soft wheat products.

## 1. Introduction

Common wheat (*Triticum aestivum* L.) is the most important cereal crop globally, serving as a major source of nutrition by providing approximately 20% of the caloric intake for humans. Due to its high starch content and unique gluten proteins present in the grain kernel [1], it is also widely processed into a variety of food products. Among the protein components, gluten proteins, specifically gliadin and glutenin, constitute approximately 85% of the total grain crude protein and play a crucial role in determining the functional properties of wheat. While individual gliadin proteins are thought to contribute minimally to the viscoelastic properties of dough, the total content of gliadin and the ratio of glutenin to gliadin have been shown to significantly influence dough characteristics, particularly dough extensibility [2,3,4]. In contrast, glutenin is characterized as a polymerized protein with a larger molecular weight, stabilized through interchain disulfide (SS) bonds. It is capable of forming a continuous gluten network and serves as the structural backbone of dough- and flour-based end products [1]. Within glutenin, the high-molecular-weight glutenin subunit (HMW-GS) and low-molecular-weight glutenin subunit (LMW-GS) constitute approximately 10% and 40%, respectively. HMW-GSs are encoded by the *Glu-A1*, *Glu-B1*, and *Glu-D1* loci located on the long arms of chromosomes 1A, 1B, and 1D, respectively. At each locus, two closely linked genes encode an x-type subunit with a higher molecular weight and a y-type subunit with a lower molecular weight [1]. Numerous studies have assessed the impact of alleles at the *Glu-1* loci on dough characteristics and bread-making quality in hard wheat [5,6,7]. The x-type HMW-GSs are considered more significant than their y-type counterparts due to their advantages in both molecular weight and quantity [8].

However, the impact of HMW-GSs on the quality of soft wheat was rarely documented. This may be attributed to the low protein content and processing applicability of soft wheat, which is typically utilized for producing cookies, cakes, and pastries [9,10]. The limited available literature indicates that allelic variation in HMW-GSs significantly influences dough properties and baking quality in soft wheat [10,11,12,13,14]. The optimal composition for soft wheat appears to involve a combination of HMW-GSs with Glu-A1c (null), Glu-B1b (7x + 8y), and Glu-D1a (2x + 12y), along with an absence of secalins [10]. In comparison to Glu-D1d (5x + 10y), Glu-D1a (2x + 12y) was associated with a shorter mixograph peak time and had a positive effect on cake quality, although it did not influence cookie quality [11]. In eastern U.S. soft winter wheat varieties, a rare subunit, Glu-D1 (2x + 121y), has been identified as desirable for producing large-diameter cookies [13]. It is evident that high-quality soft wheat generally yields flour with low water absorption while developing less tenacious but more extensible doughs [9,15]. To enhance the dough properties and baking quality of soft wheat, some researchers have developed null alleles for HMW-GSs, which undoubtedly reduce dough strength while often improving cookie quality, primarily regarding cookie diameter [16,17,18]. The deterioration of the gluten network led to a significant decrease in dough extensibility, consequently impairing the quality of sponge cakes [16,17,18]. These findings demonstrate that distinct soft wheat products exhibit varying demands for dough characteristics. However, the cookie test has been widely adopted for soft wheat quality assessment due to its high-throughput processing, excellent repeatability, and its ability to effectively evaluate key quality parameters such as water absorption and gluten properties, including strength and extensibility.

The functionality of wheat is predominantly influenced by grain protein concentration, which is significantly affected by environmental factors such as nitrogen fertilization (N) management [19,20,21,22]. Varying levels of N input significantly affect wheat quality, primarily due to alterations in the quantity of gluten protein fractions and flour water absorption [19,23]. However, a notable contradiction exists between achieving high grain yield and maintaining high quality in soft wheat production. N application typically enhances above-ground biomass, grain productivity, and grain protein content simultaneously [21,22]. To reconcile the objectives of yield and quality in soft wheat cultivation, two primary strategies are recommended: reducing N input and identifying an appropriate fertilization regime [21,22]. Consequently, the synergistic enhancement of both grain yield and quality remains a paramount goal within soft wheat breeding programs and overall wheat production.

We previously developed a set of near isogenic lines (NILs), each featuring an individual high-molecular-weight glutenin subunit (HMW-GS) null allele in the background of the soft wheat Ningmai 9. All single HMW-GS null alleles resulted in reduced dough elasticity and increased cookie diameter at the N fertilizer rate of 180 kg ha^−1^ [3], which was the recommended N rate for high-quality soft wheat production with a yield penalty [21,22]. Notably, an NIL with the *1Bx7null* allele demonstrated a significant enhancement in dough extensibility [3]. This suggested that the *1Bx7null* allele may represent a promising candidate for incorporation into soft wheat breeding programs aimed at balancing dough properties and grain yield because good dough extensibility accompanied by low dough tenacity is particularly important for end-use quality of soft wheat, such as sugar snap cookies [15]. Therefore, the objectives of this study are as follows: (1) to investigate changes in grain yield, quality parameters, and quantitative fractions of gluten proteins in both the wild type Ningmai 9 (WT) and its NIL with *1Bx7null* under varying nitrogen inputs; (2) to elucidate the contribution of *1Bx7null* in balancing end-use quality and grain yield in the field condition; and (3) to dissect the DNA sequence of *1Bx7null* and develop breeding-friendly markers associated with it. The results will provide novel insights into balancing grain yield with cookie-baking quality in soft wheat through manipulation of HMW-GS composition. 

## 2. Materials and Methods

### 2.1. Field Trials and Plant Materials

**Figure 1:**
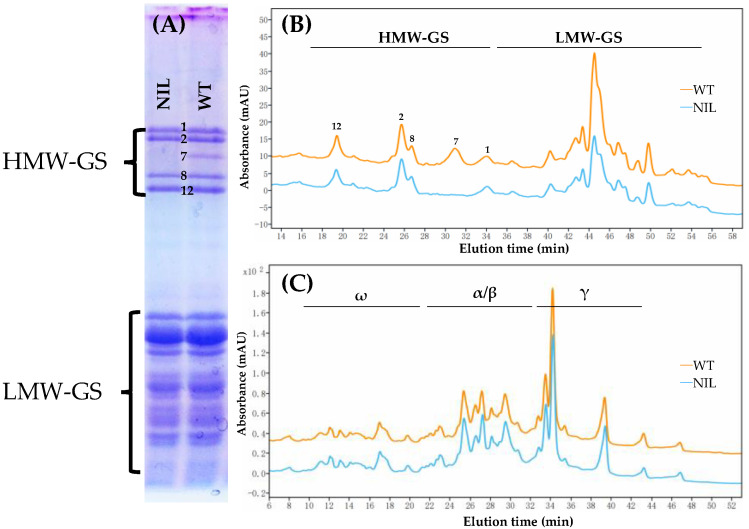
SDS-PAGE analysis of glutenin (**A**), RP-HPLC profiles of glutenin (**B**) and gliadin (**C**) in Ningmai 9 WT and its NIL.

The field trial was conducted over two consecutive growing seasons from 2021 to 2023 in Nanjing Experiment Station of Jiangsu Academy of Agricultural Sciences in Nanjing, China. This experiment station was representative for soft red winter wheat production in the middle and lower reaches of the Yangtze River in southern China [9,20]. Two genotypes were grown under two N application rates. The two genotypes were Ningmai 9 WT with the allele *1Bx7* (WT) and its near isogenic line (NIL) carrying the allele *1Bx7null* (NIL), as detailed in Table 1 and Figure 1. The WT is a soft red winter wheat exhibiting spring growth habits. The NIL was developed through successive backcrossing to incorporate the *1Bx7null* allele into the WT genetic background [3]. *1Bx7null* was a nonsense mutation allele of *1Bx7*, which was obtained through chemical mutagenesis treatment with Ethylmethane Sulfonate (EMS) in the genetic background of Ningmai 9 WT. In each growing season, both P_2_O_5_ and K_2_O were applied at a rate of 120 kg ha^−1^ as basal fertilizer. The two N application rates consisted of low nitrogen treatment at 180 kg ha^−1^ (LN) and high nitrogen treatment at 240 kg ha^−1^ (HN). Nitrogen fertilizer was applied according to a regime comprising three stages: specifically, applying 50% at sowing, followed by an additional application of 20% at the tillering stage, and concluding with another application of 30% at the stem elongation stage. Four field treatment combinations, based on genotype and nitrogen rate, were arranged in a randomized complete block design with three replicates. Each replicate plot (6.67 m^2^) consisted of five rows spaced 26.7 cm apart. The sowing density for each replicate was standardized at 2.25 × 10^6^ ha^−1^.

In each growing season, all plots were irrigated with sprinklers immediately after sowing and subsequently relied on rainfall throughout the entire growth period. Local agricultural practices were applied during cultivation. All genotypes were grown and matured well in each growing season. Key agronomic traits such as plant height, grain yield, spike density per planting area, grain number per spike, and thousand kernel weight were recorded. The total counts of spikes in the second row of each plot were recorded and converted into spikes per unit area for analysis. Additionally, twenty spikes were randomly selected from the center of each plot to assess both grain number per spike and plant height. Thousand kernel weight was determined manually post-harvest.

### 2.2. Quality Trait Testing

The AACC international methods [24] were utilized to assess the physicochemical quality traits of wheat samples. Grain hardness and moisture content were evaluated using the Single Kernel Characterization System 4100 (Perten Instruments, Springfield, IL, USA) according to AACC method 55-31.01. The measurements for grain protein, flour protein, flour moisture, and ash content were conducted employing the NIR method with the Perten DA7200 (Perten Instruments, Reno, NV, USA). Wheat kernel samples were tempered to a moisture content of 14.0% and subsequently milled into flour utilizing a Quadrumat Senior mill (Branbender GmbH & Co. KG, Duisburg, Germany), following AACC method 26-50.01. The gluten content and gluten index were determined using a Glutomatic 2200 apparatus (Perten Instruments AB, Huddinge, Sweden) according to AACC 38-12.02. Solvent retention capacity, farinograph parameters, alveograph parameters, and mixograph parameters were assessed according to the approved AACC methods 56-11.02, 54-21.02, 54-30.02, and 54-40.02, respectively. SDS-sedimentation volume was tested using 1 g flour according to Peña et al. [25]. All of the quality traits were measured with two technical replicates.

### 2.3. Baking Quality Test

The quality of sugar snap cookie was assessed according to the AACC-approved method 10-52.02. The width (W) and thickness (T) of two cookies were measured, and the cookie spread factor was calculated by dividing the width by the thickness, denoted as W/T. The cookie baking test was performed with two technical replicates. The texture profile characteristics of the cookies were evaluated using an FTC TMS-PRO Texture Analyzer (Food Technology Co., Ltd, Sterling, VA, USA), equipped with a three-point bending test module utilizing an L3PB probe and a 250 N load cell. The fixed distance between the two points was set at 6 cm. The pre-test speed and test speed were established at 1.0 mm s^−1^ and 0.5 mm s^−1^, respectively, and the initial force was calibrated to 0.5 N. Measurements recorded included maximum shear force, work associated with maximum shear force, and crispness. Crispness was quantified as the slope from the point of initial force application to that of maximum force at which cookie breakage occurred. Four cookies from each flour sample were subjected to texture analyzer testing.

### 2.4. Separation and Quantitation of Gluten Protein by SDS-PAGE and RP-HPLC

The composition of HMW-GSs in WT and NIL was separated and identified using the SDS-polyacrylamide gel electrophoresis method (SDS-PAGE) following the protocols established by Liu et al. [26]. Gliadins were isolated utilizing reversed-phase high-performance liquid chromatography (RP-HPLC), as described by Zhang et al. [2]. The quantities of individual HMW-GSs and total low-molecular-weight glutenin subunits (LMW-GSs) were also determined through RP-HPLC according to Zhang et al. [2]. The percentage of SDS-unextractable polymeric protein (%UPP) was quantified via size-exclusion high-performance liquid chromatography (SE-HPLC), as outlined by Larroque et al. [27]. The HPLC analysis system employed was a Dionex Ultimate 3000 (Thermo Scientific, Waltham, MA, USA) equipped with UV detection at 214 nm. CHROMELEON 6.80 manager software facilitated control over the HPLC system and enabled quantification of the elution peaks corresponding to protein fractions in the chromatograms.

### 2.5. Nucleotide Sequence Analysis of 1Bx7 and 1Bx7null

The genomic DNAs of WT and NIL were extracted from wheat seedlings using the TIANGEN^®^ DNAsecure Plant Kit (Tiangen Biochemical Technology Co. (Beijing), Ltd., Beijing, China), employing a column-based method. Two full-length gene sequences, *1Bx7* and *1Bx7null*, were PCR-amplified with specifically designed primers based on published sequences [28]. The resulting sequences were aligned using DNAMAN version 6.0 (Lynnon, Quebec, Canada). The primers used included a forward primer, 5′-TCTTCTCACCTTTCTTCATAGG C-3′, and a reverse primer, 5′-TTCCCTTGCTTGGATGATGGTAG-3′ (Figure S1). The PCR reaction mixture was prepared in a total volume of 50 µL and contained the following components: 2 µL genomic DNA (50 ng), 10 µL of 5×PrimeSTAR Buffer (Mg^2+^ Plus), 4 µL dNTP (2.5 mM), 2 µL forward primer (10 µM), 2 µL reverse primer (10 µM), 0.5 µL PrimeSTAR HS polymerase (2.5 U/µL), and 29.5 µL ddH_2_O. The thermal cycling conditions for the PCR included an initial denaturation step at 95 °C for 3 min, followed by 35 cycles consisting of denaturation at 94 °C for 30 s, annealing at 64 °C for 15 s, and extension at 72 °C for 30 s; this was concluded with a final extension step at 72 °C for an additional duration of 10 min. Amplification products were resolved on agarose gels containing a concentration of approximately 1.5% (wt/vol).

After gel purification, the amplified specific band was cloned into pEASY-Blunt cloning vector and subsequently transformed into *E.coli* DH5α competent cells, which were cultured at 37 °C for 14 h. Positive clones were identified through PCR using the *E. coli* cultures as templates. Four positive clones from each PCR product were sequenced at Shanghai Sangon Biological Engineering & Technology and Service Co., Ltd., Shanghai, China. The full-length sequences (5′-3′) of *1Bx7* and *1Bx7null* were assembled using DNAMAN 6.0 (Lynnon, QC, Canada).

### 2.6. RNA Extraction and Quantitative Reverse Transcription-PCR (qRT-PCR)

Six wheat kernels from the central region of each spike were collected from both WT and its NIL at 7-day intervals post-anthesis (DPAs). Total RNA was extracted from the collected kernels using the SV Total RNA Isolation System (Promega, Beijing, China). First-strand cDNA synthesis was performed utilizing the TaKaRa One Step RNA PCR Kit (AMV) (Takara Bio, Shanghai, China). Fluorescent quantitative real-time PCR (FQ-PCR) was conducted using a LightCycler^®^480 II Real-Time PCR System (Roche, Meylan, France), with a 20 µL reaction mixture comprising the following: 10 µL 2×SYBR Premix EX Taq (Takara Bio, Dalian, China), 0.8 µL forward primer (10 µM), 0.8 µL reverse primer (10 µM), 1 µL of the first-strand cDNA template, and 7.4 µL of ddH_2_O. The FQ-PCR conditions included an initial denaturation step at 95 °C for 30 s, followed by 40 cycles consisting of denaturation at 95 °C for 5 s, annealing at 60 °C for 20 s, and extension at 72 °C for another 20 s. β-actin served as the internal control gene. The *1Bx7* gene-specific primers was picked as follows: forward 5′-TCTTTGCGGCAGTAGTCG-3′ and reverse 5′-ACATTGTAGTTGTCCAGAGGC-3′ at the starting position of the coding region (Figure S1). Three biological replicates were tested within one field replicate plot to determine the average relative expression level of *Glu-B1x*.

### 2.7. Development of Cleaved Amplified Polymorphic Sequence (CAPS) Marker

The 300 bp flanking sequences, both upstream and downstream of the single base substitution site, were input into dCAPS Finder 2.0 (http://helix.wustl.edu/dcaps/dcaps.html, accessed on 8 January 2025) to identify primers for amplifying a sequence that includes the substitution site. This analysis aimed to locate a restriction endonuclease capable of recognizing the SNP site. The finalized anchored primers are as follows: forward primer 5′-CAGCAGGGGTCATACTATCCA-3′ and reverse primer 5′-GTCCTGGCTGCTGTGAAGTT-3′. The PCR reaction mixture, totaling 25 µL, consisted of the following: 1 µL genomic DNA (20 ng), 2.5 µL 10×thermalPol^®^ reaction buffer (B9004S) (New England Biolabs (Beijing) Co., Ltd., Beijing, China), 0.5 µL dNTP (10 mM), 0.5 µL forward primer (10 µM), 0.5 µL reverse primer (10 µM), 0.125 µL Taq DNA Polymerase (5 U/µL, M0267V) (New England Biolabs (Beijing) Co., Ltd., Beijing, China), and 19.875 µL of ddH_2_O. The thermal cycling conditions for PCR included an initial denaturation step at 95 °C for 3 min, followed by 35 cycles consisting of denaturation at 94 °C for 30 s, annealing at 64 °C for 15 s, and extension at 72 °C for 30 s; the process concluded with a final extension step at 72 °C for 7 min. Subsequently, 10 µL of the PCR product was digested using 3 µL BstEII-HF mixture stock solution containing 1.3 µL rCutSmart^TM^ Buffer (B6004S) (New England Biolabs (Beijing) Co., Ltd., Beijing, China), 0.3 µL BstEII-HF (20 U/µL, R3162V), and 1.4 µL ddH_2_O. All amplification products and digestion products were resolved on agarose gels with a concentration of 2.0% (wt/vol).

### 2.8. CAPS Marker Validation

The validation of CAPS markers was conducted using nine genotypes: NIL and eight released cultivars (Table 1), along with three F_2_ populations (each comprising six individuals). SDS-PAGE analysis revealed that Ningmai 14, Ningmai 18, and Ningmai 9 WT share identical *Glu-B1* alleles. Seven genotypes with distinct alleles at the *Glu-B1* locus were utilized for the validation of CAPS markers within the natural population, including both WT and its NIL, Lumai 23, Ninghongmai 529, Wanmai 33, Glenlea, and Yangmai 22. Three F_2_ generation populations were employed for validation within the breeding program. These populations were derived from three hybrid crosses: Ningmai 14/NIL, Ningmai 18/NIL, and Yangmai 22/NIL. The genomic DNA of each genotype was extracted from a composite leaf sample consisting of five individual plants.

### 2.9. Statistical Analysis

Statistical analysis of wheat and cookie quality properties was performed using SAS version 9.4 (SAS Institute Inc., Cary, NC, USA). PROC MEANS was used to calculate the mean, standard deviation, and maximum and minimum value of each variable with different classification. The results of normality and homogeneity of variance tests confirmed that the data met the assumptions required for ANOVA analysis. Statistical variations attributable to year, genotype, nitrogen treatments, and their interactions were analyzed using PROC GLM. Year and field replicates were treated as random effect. To identify significant differences among years, genotypes, and nitrogen application rates, the least significant difference (LSD) multiple comparison test was utilized.

## 3. Results

### 3.1. Composition of Gluten Proteins in WT and Its NIL

SDS-PAGE (Figure 1A) and RP-HPLC (Figure 1B) were utilized to qualitatively separate the glutenin subunits of both WT and NIL. These two genotypes exhibit identical glutenin compositions, with the exception of the absence of the 1Bx7 in the NIL. Notably, due to its high separation efficiency, RP-HPLC (Figure 1C) distinctly demonstrated that the gliadin compositions of WT and NIL are equivalent.

### 3.2. Comparative Analysis of Glu-B1x Genes from WT and Its NIL

The full-length sequences of the *Glu-B1x* gene in both WT and NIL were amplified using a pair of specific primers flanking the coding region. The open reading frame, consisting of 2370 base pairs, was identified and contained a double stop codon (Figure S1). The sequence of the *Glu-B1x* gene in WT was found to be identical to that reported previously by Anderson and Greene [28]. In comparison to WT, the sequence of *1Bx7null* in NIL exhibited a single nucleotide substitution from C to T at position 514 bp, resulting in the alteration of the glutamine codon CAA into a stop codon TAA. The stop codons usually led to the suppression of mutated gene expression and resulted in truncated, non-functional polypeptide chains. However, based on detections of SDS-PAGE and RP-HPLC, no truncated polypeptide was presented.

### 3.3. Expression of the Glu-B1x Gene During the Grain Filling Stage

Total RNA was extracted and reverse transcribed to cDNA from the grains of both WT and NIL subjected to varying nitrogen inputs during the filling stage, sampled at 7-day intervals post-anthesis (DPAs). The expression profiles of *Glu-B1x* of the two genotypes were assessed using qRT-PCR as illustrated in Figure S2. In WT, the expression of *1Bx7* was detected at 7 DPAs, peaking with relative expression levels of 1.7451 under low nitrogen (LN) conditions and 4.8010 under high nitrogen (HN) conditions at 14 DPAs. Subsequently, these levels declined significantly to very low values, with relative expressions recorded at 0.0264 and 0.1207, respectively, by 21 DPAs. In contrast to WT, the relative expressions of *1Bx7null* remained at persistently very low levels during the entire grain filling. This was highly likely attributed to the suppression of gene expression due to a premature termination codon present in the sequence of *1Bx7null*, as illustrated in Figure S1. Herein, we observed a detectable trace transcript for the *1Bx7null* allele. Although the molecular mechanism underlying this reduction requires further investigation, one plausible explanation is that the introduced premature stop codon may target the transcript for nonsense-mediated mRNA decay (NMD).

### 3.4. ANOVA of Agronomic Traits and Wheat Quality Traits

The ANOVA results indicated that nitrogen was the most significant source of variation for plant height, spike number, and grain yield (Table S1). Additionally, the growing year had a substantial impact on spike density per planting area, grain number per spike, and yield. In contrast, genotype exhibited the least influence on plant height and yield components; this can be attributed to the fact that the two genotypes tested are near isogenic lines. The variations in plant height and yield components, particularly thousand kernel weight, were minimally affected by interactions among year, genotype, and nitrogen levels.

For kernel quality traits, genotype was the most significant source of variation. Kernel hardness and flour yield were also notably influenced by year and the interaction between year and genotype, likely due to variable rainfall during the grain filling stage, as the field trials were conducted in a rainy region typical for soft wheat production. Regarding flour quality traits, nitrogen primarily affected flour protein content, gluten content, and SDS-sedimentation volume. The gluten index was significantly influenced by both N and genotype. Lactic acid solvent retention capacity (SRC) was markedly affected by genotype and year. Sodium carbonate SRC and sucrose SRC were significantly impacted by nitrogen and year, respectively. In terms of rheological properties of dough, N represented the most critical source of variation. Genotype also played a substantial role in farinograph stability and weakness, mixograph peak time and integral values, as well as alveograph parameters. No clear pattern could be discerned regarding variations in gluten protein fractions in terms of quantity. The appearance of the cookies was predominantly determined by genotype and N levels. Cookie hardness, specifically maximum shear force, was only significantly influenced by N. However, cookie crispness proved to be more susceptible to variations and was significantly affected by year, N level, and genotype, as well as the interaction between year and N level.

### 3.5. Grain Yield and Wheat Quality Traits in WT and NIL

**Figure 2:**
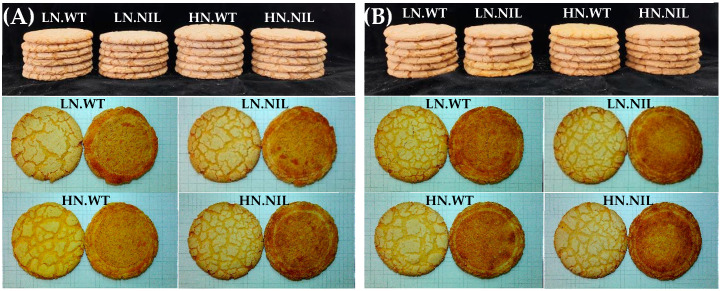
Cookies produced from Ningmai 9 WT and its NIL under varying nitrogen inputs (Note: LN—low nitrogen input; HN—high nitrogen input; (**A**) 2021–2022 growing season; (**B**) 2022–2023 growing season).

**Figure 3:**
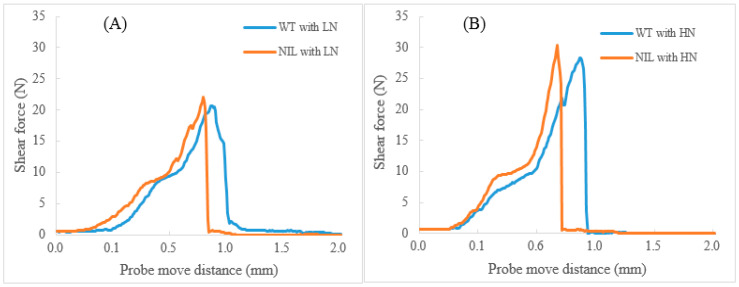
Typical texture characteristic profiles of cookies tested using a Texture Analyzer (Food Technology Co., Ltd) for Ningmai 9 WT and its NIL under varying nitrogen inputs (Note: LN (**A**)—low nitrogen input; HN (**B**)—high nitrogen input).

For kernel quality traits such as hardness, protein content, and flour yield, no significant differences were observed between WT and NIL. In terms of flour quality traits, the lactic acid SRC, SDS-sedimentation volume, and gluten index in WT were significantly higher than the corresponding values in NIL (Table S2). However, traits related with protein quantity, such as flour protein content and gluten content, exhibited similar levels between WT and NIL. All parameters associated with dough tenacity demonstrated significantly higher values in WT compared to NIL. Conversely, alveograph L, reflecting dough extensibility, was found to be greater in NIL than in WT. Although there was no significant difference observed regarding the amount of unextractable polymeric protein (UPP) between WT and NIL, a notable and significant change was seen in the relative quantity of unextractable polymeric protein (%UPP). Furthermore, WT displayed a significantly higher glutenin/gliadin ratio as well as an elevated HMW-GS/LMW-GS ratio than NIL. NIL exhibited a significantly larger cookie diameter (17.87 cm), spread ratio (11.27), and crispness (28.57 N/m) than those recorded for WT, which had corresponding measurements of 17.23 cm, 10.42, and 20.87 N/m, respectively (Table S2 and Figure 2 and Figure 3).

### 3.6. Impact of Nitrogen Application Rates on Wheat Quality Traits

In this study, N was identified as a significant source of variation for most traits (Table S1). Overall, compared to LN conditions, HN treatment markedly increased spike number, plant height, grain yield, protein content, gluten content, and various quality traits associated with dough tenacity and mixing stability (Table S2). For example, under both HN and LN conditions, the alveograph *p* values were 67.77 mm and 49.61 mm, respectively. Additionally, HN treatment resulted in a decrease in %UPP (*p* < 0.05) and glutenin/gliadin ratio (*p* > 0.05). Regarding cookie quality traits, the application of HN led to a significantly reduced cookie width; conversely, it produced a significantly higher crispness when compared to measurements from LN (Tables S2 and S3, and Figure 2 and Figure 3).

**Figure 4:**
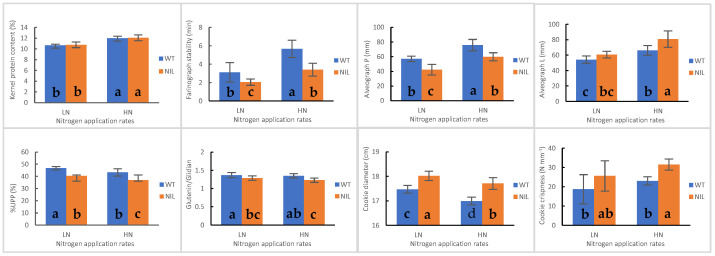
Variations in main flour and quality traits of WT and NIL under varying nitrogen inputs (Note: Different lowercase letters within a bar chart denote significance at *p* < 0.05.).

Specifically, nitrogen application rate exerted comparable effects on key dough characteristics and cookie quality between WT and NIL (Figure 4). Under HN input, however, significant differences in the alveograph L and cookie crispness were observed between WT and NIL, in contrast to LN input conditions. The NIL+LN treatment yielded the largest cookie diameter at an impressive measurement of 18.02 cm, followed by NIL+HN, WT+LN, and WT+HN in that order. In addition to yield improvement, the NIL under HN conditions demonstrated significantly superior cookie quality compared to the WT under LN conditions (Figure 4 and Table S3).

### 3.7. Development and Validation of CAPS Marker for 1Bx7null

**Figure 5:**
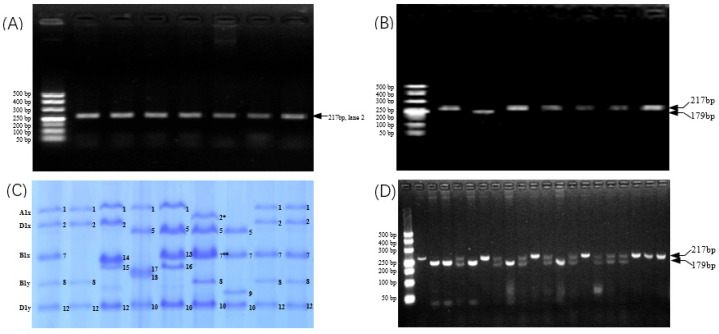
(**A**) PCR products amplified by the designed CAPS marker at *Glu-B1* loci in nine genotypes exhibiting different allelic variations (Note: From left to right, DL 500 bp Marker, Ningmai 9 WT, Ningmai 9 NIL, Lumai 23, Ninghongmai 529, Wanmai 33, Glenlea, Yangmai 22). (**B**) Digested PCR products amplified by the designed CAPS marker at *Glu-B1* loci in nine genotypes with varying allelic variations (Note: From left to right, DL 500 bp Marker, Ningmai 9 WT, Ningmai 9 NIL, Lumai 23, Ninghongmai 529, Wanmai 33, Glenlea, Yangmai 22). (**C**) SDS-PAGE profiles of HMW-GS in genotypes displaying different allelic variations at *Glu-B1* loci (Note: From left to right, Ningmai 9 WT, Ningmai 9 NIL, Lumai 23, Ninghongmai 529, Wanmai 33, Glenlea, Yangmai 22, Ningmai 14, Ningmai 18). (**D**) Digested PCR products amplified by the designed CAPS marker from F_2_ generation populations derived from three wheat hybrids (Note: From left to right, lane 1: DL 500 bp Marker; lane 2: Ningmai 9 WT; lane 3: Ningmai 9 NIL; lanes 4–9: F_2_ populations of Ningmai 14/Ningmai 9 NIL; lanes 10–15: F_2_ populations of Ningmai 18/Ningmai 9 NIL; lanes 16–21: F_2_ populations of Yangmai 22/Ningmai 9 NIL.

As previously described, a single base substitution was identified at 514 bp in the coding sequence (CDS) of *1Bx7null* compared to *1Bx7*. Utilizing the dCAPS Finder 2.0 software, a pair of primers was selected: the forward primer 5′-CAGCAGGGGTCATACTATCCA-3′ and the reverse primer 5′-GTCCTGGCTGCTGTGAAGTT-3′. This specific primer pair successfully amplified a fragment of 217 bp that included the target single base in both WT and NIL, as demonstrated by lanes 2 and 3 in Figure 5A. Furthermore, using BstEII endonuclease, the amplified fragment from NIL could be specifically digested into two fragments measuring 179 bp and 38 bp at the recognition sequence of 5′-G↓GTNACC-3′, as shown in lane 3 of Figure 5B.

To assess the capability of the developed CAPS marker in distinguishing *1Bx7null* from *1Bx7* and other alleles of *Glu-B1x* present in natural germplasms, genomic DNA was extracted from five varieties characterized by distinct alleles: (*Glu-B1h* (14x + 15y), Lumai 23); (*Glu-B1i* (17x + 18y), Ninghongmai 529); (*Glu-B1g* (13x + 19y), Wanmai 33); (*Glu-B1al* (7^OE^ + 8), Glenlea); and (*Glu-B1c* (7x + 9y), Yangmai 22). These samples were subjected to PCR amplification followed by BstEII digestion, as detailed in Table 1 and illustrated in Figure 5C from lane 3 to lane 7. The results indicated that the developed markers successfully amplified fragments of similar sizes across these five varieties, as shown in Figure 5A, lanes 4 to 8. Furthermore, these amplified products could not be digested by BstEII, as demonstrated in Figure 5B, lanes 4 to 8.

Three F_2_ populations were further utilized to validate whether the developed CAPS marker could effectively identify the *1Bx7null* allele within breeding populations. Herein, all male parents, namely Ningmai 14, Ningmai 18, and Yangmai 22, represented important germplasm resources for soft wheat breeding (Table 1, Figure 5C). The alleles of both *Glu-B1b* (7x + 8y) and *Glu-B1c* (7x + 9y) present in these parental lines are among the most prevalent alleles, particularly in the Yangtze River basin of China. Both homozygous and heterozygous forms of *1Bx7* and *1Bx7null* were distinctly identifiable (Figure 5D from lane 4 to lane 21). The low-density bands observed in the heterozygotes resulted from a reduced concentration of PCR products, wherein both target sequences corresponding to *1Bx7* and *1Bx7null* were amplified simultaneously.

## 4. Discussion

### 4.1. Investigation of the Genetic Background of Ningmai 9 WT and Its NIL

In the same field under a consistent fertilizer regime, the phenotypic traits of Ningmai 9 WT and its NIL were evaluated. Observations regarding plant height, leaf, and leaf sheath color, days to heading, as well as other characteristics such as flag leaf angle and spike morphology, indicated a genetic similarity between the two lines. The SDS-PAGE patterns and RP-HPLC profiles further corroborated their shared genetic background. Notably, the high-resolution RP-HPLC elution profiles of gliadin were identical for both genotypes, which is recognized as a distinctive fingerprint for wheat genotypes [29]. Consequently, this pair of NILs serves as an ideal model for investigating the impact of the 1Bx7 subunit on grain yield and wheat functionalities. However, the NIL was developed through only five backcrosses and might possess small introgressed segments from the donor parent.

### 4.2. The Variation in Grain Yield and Quality Under Variable N Inputs

The area where this study was conducted is recognized as the most suitable region for high-quality soft wheat cultivation in China [20]. However, two environments (two years, one location) were still limited, and additional environmental factors, such as soil and rainfall, may influence the existing conclusions. Currently, two national standards have been established in China: GB/T 17893-1999 [30] and GB/T 17320-2013 [31]. GB/T 17893-1999, titled “High Quality Wheat—Weak Gluten Wheat”, serves to define high-quality soft wheat for trade and processing applications and is characterized with kernel crude protein content ≤ 11.5% (on a dry basis), wet gluten content ≤ 22% (14% wet basis), and farinograph stability ≤ 2.5 min; such characteristics make it suitable for cookie and cake production. Meanwhile, GB/T17320-2013, titled “Quality Classification of Wheat Varieties”, is used to classify various wheat varieties by quality and is recognized with corresponding limit values set at <12.5%, <26%, and <3.0 min, respectively, additionally requiring farinograph absorption to be <56% and Zeleny sedimentation volume to be <30 mL. However, achieving both increased grain yield and compliance with end-use quality requirements are critical in China [21,32].

In actual field production, the conventional nitrogen (N) application rate in China typically ranges from 240 to 270 kg ha^−1^ [22]. At this level of N application, it becomes challenging to balance grain yield and quality in soft wheat, as all quality traits related to protein content are highly susceptible to nitrogen fertilizers [22]. According to a report by Hu et al. [33], fewer than 1.0% of samples produced from soft wheat varieties met the requirements outlined in GB/T 17893-1999. This finding was further corroborated by Zhang et al. [15,34,35], who indicated that when employing conventional field practices and approved high-quality soft wheat varieties, all grain samples exhibited excessive protein content and gluten strength compared to the two national standards, although grain hardness and flour water absorption met the criteria in GB/T17320-2013. To achieve processing applicability, it is necessary for the N application rate to be below what is required for optimal yield [21,22]. Consequently, a N application rate of less than 200 kg ha^−1^ has been recommended for producing high-quality soft wheat in this region [21,22], which inevitably results in reduced grain yield. As confirmed by this study, the average grain yield at a N application rate of 240 kg ha^−1^ was found to be 16.4% higher than that at a rate of 180 kg ha^−1^. Furthermore, average kernel protein content increased significantly by 12.23%, while alveograph P and farinograph stability rose by approximately 33.8% and 76.3%, respectively. With elevated nitrogen input levels, the cookie diameters for Ningmai 9 WT and its NIL were significantly reduced by approximately 2.74% and 1.72%, respectively (Table S3).

Additionally, the dough tenacity and extensibility of soft wheat in China do not meet the criteria as suggested by CIMMYT or USDA. These criteria particularly emphasize good milling quality—characterized by low kernel hardness, high flour yield, and a favorable soft equivalent—as well as low dough strength and water absorption, high dough extensibility, and large cookie diameter, while relaxing the requirement for kernel protein content [9,15,36,37]. Numerous studies have indicated that protein content was negatively and significantly correlated with cookie diameter [38,39]. However, some research has also concluded that there is a weak or ambiguous correlation between protein content and cookie-making quality, especially under conventional nitrogen application rates [2,15,34,39,40]. The differing conclusions may be attributed to two factors: first, the genetic diversity of experimental materials including variations in gluten protein composition, kernel hardness, and pentosan content; and second, the sample size along with the variation range of protein content among the experiment materials. As recently reported [35], seven out of fifteen varieties exhibited higher protein content alongside larger cookie diameters compared to Ningmai 9. This suggests that protein quality may be more critical than protein quantity in soft wheat, a notion supported by previous reports [12,13,14]. However, it is evident that low flour water absorption coupled with weaker yet more extensible dough are essential attributes for ensuring processing applicability in high-quality soft wheat used for cookies. In soft wheat breeding programs, protein content and kernel hardness may serve as fundamental traits. Superior cookie baking performance can be achieved through the reduction in kernel hardness, damaged starch, and pentosan content alongside modified dough quality [15,41].

### 4.3. Effect of 1Bx7null on Dough Properties and Baking Quality

A number of studies have indicated that the dough strength and baking quality of soft wheat can be enhanced by assembling alleles at *Glu-1* loci. The primary strategy involves utilizing a limited weak-gluten-related HMW-GS or the knockout of genes encoding HMW-GS [10,11]. Nevertheless, one report demonstrated that the deletion of allele *1Dy12* did not enhance cookie baking quality. This outcome was primarily attributed to the high kernel hardness and protein content observed in both the control and its mutant lines [42]. Additionally, our previous findings revealed significant variations in dough strength and cookie baking quality among mutation lines with identical HMW-GS deletions [43]. This suggests that the functional impact of HMW-GS alleles is significantly influenced by genetic background. Moreover, the previous report indicated that the inferior gluten quality resulting from the deletions of HMW-GSs was detrimental to cake quality [17].

For dough extensibility, most genotypes tested with HMW-GS null exhibited reduced dough extensibility [7,16,44,45]. A few hard kernel texture lines with specific HMW-GS deletions demonstrated unchanged or even increased dough extensibility, which contributed to enhanced quality of steamed bread and tortillas [4,7,44,45]. Our previous studies indicated that under low nitrogen input of 180 kg ha^−1^, the NIL with *1Bx7null* could simultaneously increase dough extensibility while decreasing dough tenacity [3]. Nevertheless, the response of this genotype to high nitrogen input remains unclear, an aspect that is particularly crucial for its application in soft wheat breeding programs and field production. In the present study, we further identified that the NIL showed greater dough extensibility under HN input compared to LN input (Figure 4, Table S3). This observation can be attributed to the significant reduction in the glutenin/gliadin ratio in NIL, as gliadin primarily contributes to the extensibility of wheat dough [2,19]. On the one hand, gliadin is particularly sensitive to environmental fluctuations and tends to increase more rapidly than glutenin with rising protein content [23,46,47]; on the other hand, in the NIL with *1Bx7null*, the possible compensatory increase in alcohol-soluble proteins further increases its percentage [18].

For baking quality, as anticipated, the NIL with *1Bx7null* produced cookies that were larger and crisper than those of the WT under both LN and HN conditions (Figure 4 and Table S3). This observation can be attributed to its weaker and softer dough characteristics, fundamentally due to its lower % UPP and Glutenin/Gliadin ratio, which aligns with findings reported by Zhang et al. [16] and Zheng et al. [40]. Furthermore, an increase in flour protein content significantly enhanced cookie firmness and crispness (Figure 4 and Tables S2 and S3). This result is consistent with previous studies conducted by Moiraghi et al. [39] and Zheng et al. [40].

### 4.4. Development and Validation of CAPS Marker

Ningmai 9 WT was the first soft red winter wheat variety released in China, specifically suitable for cookie and cake processing. However, Ningmai 9 exhibited relatively higher kernel hardness, pentosan content, and starch pasting properties (SRC) as well as alveograph P and P/L values compared to some later-released varieties; notably, its P/L value typically hovered around 1.0 [3,15]. In this study, the dough strength and extensibility of Ningmai 9 was genetically enhanced by incorporating the allele *1Bx7null*. Under a HN input of 240 kg ha^−1^, the cookie diameter of NIL with the *1Bx7null* allele was comparable to that of WT with the *1Bx7* allele under a LN input of 180 kg ha^−1^ (Figure 4). This finding suggests that the *1Bx7null* allele holds promise for application in soft wheat breeding programs. However, all genes encoding HMW-GSs were dominant except for the naturally silenced *1Ay* gene [1]. Consequently, the SDS-PAGE method may not be feasible for differentiating between *1Bx7null* and *1Bx7* in heterozygous individuals or strains. We successfully created a codominant CAPS marker with the assistance of BstEII restriction endonuclease. This codominant CAPS marker developed in this study exhibited strong efficacy in differentiating between *1Bx7* and *1Bx7null* and can also differentiate *1Bx7null* from the other *Glu-B1* alleles. Thus, accurate marker-assisted selection for *1Bx7null* can be conducted in soft wheat breeding programs.

## 5. Conclusions

In the genetic background of soft wheat Ningmai 9, the *1Bx7null* allele was identified to improve cookie quality by enhancing dough extensibility while reducing dough tenacity under both LN and HN input conditions. Accurate marker-assisted selection for *1Bx7null* can be conducted using the newly developed CAPS marker. Future soft wheat varieties incorporating the *1Bx7null* allele are anticipated to achieve an improved equilibrium between grain yield and end-use quality. Future studies will examine the impact of *1Bx7null* on soft wheat products beyond cookies and its effects across diverse parental backgrounds.

## Figures and Tables

**Table 1:** HMW-GS compositions of NIL and eight varieties used for CAPS marker validation.

Genotype	Glu-A1x	Glu-B1x	Glu-B1y	Glu-D1x	Glu-D1y
NIL	1	Null	8	2	12
WT	1	7	8	2	12
Lumai 23	1	14	15	2	12
Ninghongmai 529	1	17	18	5	10
Wanmai 33	1	13	19	5	10
Glenlea	2^*^	7^OE^	8	5	10
Yangmai 22	0	7	9	5	10
Ningmai 14	1	7	8	2	12
Ningmai 18	1	7	8	2	12

## Data Availability

The original contributions presented in the study are included in the article/Supplementary Materials, further inquiries can be directed to the corresponding author.

## Supplementary Materials

The following supporting information can be downloaded at https://www.mdpi.com/article/10.3390/foods14234137/s1. Figure S1: Full-length gene sequences (5′-3′) of *B1x7* and *B1x7null*, cloned from Ningmai 9 WT and its NIL, respectively; Figure S2: Relative express levels of *Glu-B1x* in Ningmai 9 WT and its NIL under varying nitrogen inputs during the kernel filling stage; Table S1: Analysis of Variance (ANOVA) for yield traits and quality traits; Table S2: Comparison of yield traits and quality traits across years, genotypes, and nitrogen application rates; Table S3: Comparison of yield traits and quality traits among combined treatments of genotype and nitrogen application rates.

## Abbreviations

The following abbreviations are used in this manuscript:

HMW-GS	High-molecular-weight glutenin subunit
HMW-GS	Low-molecular-weight glutenin subunit
LN	Low nitrogen
HN	High nitrogen
SNP	Single nucleotide polymorphism
WT	Wild type
NIL	Near isogenic line
CAPS	Cleaved amplified polymorphic sequence
CDS	Coding DNA sequence
NMD	Nonsense-mediated mRNA decay
