# Supplementary material for: Effect of 1Bx7null on Soft Wheat Cookie Quality Under Different Nitrogen Inputs and Its CAPS Marker Development
Source: Foods. 2025 Dec 2;14(23):4137. doi: 10.3390/foods14234137 (PMC12691852; doi:10.3390/foods14234137)
Supplement: Supplementary file 1 [file foods-14-04137-s001.zip › foods-3962955-supplementary/Supplementary Files_revised/Figure S1,S2_Revised.pdf]

-173 TCTTCTCACGCTTTCTTCATAGGCATAGGCTAAACTAACCTCGGCGTGCACACAACCATGTCCTGAACCTTCA

-100 CCTCGTCCCTATAAAAGCCCATCCAACCTTCACAATCTCATCATCACCCACAACACCGAGCACCCCAATCTACAGATCAATTCACTGACAGTTCACTGAG

1 **ATG**GCTAAGCGCCTGGTCCTCTTTGCGGCAGTAGTCGTCGCCCTCGTGGCTCTCACCGCCGCTGAAGGTGAGGCCTCTGGACAACTACAATGTGAGCACG

101 AGCTCGAGGCATGCCAACAGGTGGTGGACCAGCAACTCCGAGACGTTAGCCCCGGGTGCCGCCCCATCACCGTCAGCCCGGGCACGAGACAATACGAGCA

201 GCAACCTGTGGTGCCGTCCAAGGCCGGATCCTTCTACCCCAGCGAGACTACGCCTTCGCAGCAACTCCAACAAATGATATTTTGGGGAATACCTGCACTA

301 CTAAGAAGGTATTACCCAAGTGTAACTTCTTCGCAGCAGGGGTCATACTATCCAGGCCAAGCTTCTCCCCAACAGTCAGGACAAGGACAGCAGCCAGGAC

401 AAGAACAGCAACCAGGACAAGGGCAACAAGATCAGCAGCCAGGACAAAGACAACAAGGATACTACCCAACTTCTCCGCAACAGCCAGGACAAGGGCAACA

501 ACTGGGACAAGGGCAACCAGGGCACTACCCAACTTCACAGCAGCCAGGACAAAAGCAGCAGGCAGGACAAGGGCAACAATCAGGACAAGGACAACAAGGG ***Bx7***

501 ACTGGGACAAGGGTAACCAGGGTACTACCCAACTTCACAGCAGCCAGGACAAAAGCAGCAGGCAGGACAAGGGCAACAATCAGGACAAGGACAACAAGGG ***Bx7null***

601 TACTACCCAACTTCCCCGCAACAGTCAGGACAAGGGCAACAACCGGGACAAGGGCAACCAGGGTACTACCCAACTTCTCCGCAGCAGTCAGGACAATGGC

701 AGCAACCAGGACAAGGGCAACAACCAGGACAAGGGCAGCAATCAGGACAAGGGCAACAAGGTCAGCAGCCAGGACAAGGGCAACGACCAGGACAAGGACA

801 ACAAGGGTACTACCCAATTTCTCCGCAACAGCCGGGACAAGGGCAACAATCAGGACAAGGGCAACCAGGGTACTACCCAACTTCTTTGCGGCAGCCAGGA

901 CAATGGCAGCAACCAGGACAAGGGCAGCAACCAGGACAAGGGCAACAAGGTCAGCAGCCAGGACAAGGACAACAATCAGGACAAGGACAACAAGGATACT

1001 ACCCAACTTCTCTGCAACAGCCAGGACAAGGGCAACAACTGGGACAAGGGCAACCAGGGTACTACCCAACTTCGCAGCAGTCGGAACAAGGGCAGCAGCC

1101 AGGACAAGGAAAACAACCAGGACAAGGACAACAAGGGTACTACCCAACTTCTCCGCAACAGTCAGGACAAGGGCAACAACTGGGACAAGGGCAACCAGGG

1201 TACTACCCAACTTCTCCACAGCAGTCAGGACAAGGACAACAATCAGGACAAGGACAACAAGGGTACTACCCAACTTCTCCGCAACAGTCAGGACAAGGGC

1301 AACAACCGGGACAAGGGCAATCGGGGTACTTCCCAACTTCTCGGCAGCAGTCAGGACAAGGGCAGCAGCCAGGACAAGGACAACAGTCGGGACAAGGGCA

1401 ACAAGGTCAGCAACCAGGACAAGGACAACAAGCGTACTACCCAACTTCTTCGCAACAGTCAAGACAAAGGCAACAGGCAGGACAATGGCAACGACCGGGA

1501 CAAGGGCAACCAGGGTACTACCCAACCTCTCCACAGCAGCCAGGACAAGAGCAACAATCAGGACAAGCGCAACAATCAGGACAATGGCAACTAGTGTACT

1601 ACCCAACTTCTCCGCAACAGCCAGGCCAATTGCAACAACCAGCACAAGGGCAACAACCAGCACAAGGGCAACAATCAGCACAAGAGCAACAGCCAGGACA

1701 AGCGCAACAATCAGGACAATGGCAACTAGTGTACTACCCAACTTCTCCGCAACAGCCAGGACAATTGCAACAACCAGCACAAGGGCAACAAGGGTACTAC

1801 CCAACTTCTCCACAACAGTCAGGACAAGGGCAACAAGGGTACTACCCAACTTCTCCGCAACAGTCAGGACAAGGGCAACAAGGGTACTACCCAACTTCTC

1901 CGCAACAGTCAGGACAAGGGCAGCAGCCAGGACAAGGACAACAGCCAAGACAAGGGCAACAAGGGTACTACCCAATTTCTCCGCAGCAGTCAGGACAAGG

2001 GCAACAACCAGGACAAGGGCAACAAGGATACTACCCAACTTCTCCGCAGCAGTCAGGACAAGGGCAACAACCAGGACATGAGCAACAGCCAGGACAATGG

2101 CTGCAACCAGGACAAGGGCAACAAGGGTACTATCCAACTTCTTCACAGCAGTCAGGACAAGGGCATCAATCAGGACAAGGGCAACAAGGGTACTACCCAA

2201 CTTCTCTGTGGCAACCAGGACAAGGGCAACAAGGCTACGCCAGCCCATACCATGTTAGCGCGGAGTACCAGGCGGCCCGCCTAAAGGTGGCAAAGGCGCA

2301 GCAGCTCGCGGCACAGCTGCCGGCAATGTGCCGGCTGGAGGGCAGCGACGCATTGTCGACCAGGCAG**TGATAG**AACTCTCTGCAGCTTGCATGGTGCTTG

2401 GGCATGCATGCACCTTAGCTATACAATAAACGTGACGTGTGTTCACAGCTTTTCGTGTAACTAGAGTAAAGCCCAATAATAATGCAAGATGAAAAGCTTC

2501 TCCGACTAAAAAAAGAACAAAACTGGTGCTATATAGTATGTGTTGCATGTCTCAGTTCATTGTCAGCCACTGAATGCAAACATACTCCTATATTTTGCAT

2601 GCGAAAAAAGAGGGTGTGGCAATATCCCTAGCTAAAACTAACAACAGTTAGAAGGAAAAAAGAACAGACCAAAAATGGCTTCTTGGTTTGAGACCTACCA

2701 TCATCCAAGCAAGGGAA

Figure S1. Full-length gene sequences (5'-3') of *B1x7* and *B1x7null*, cloned from Ningmai 9 WT and its NIL, respectively (Note: TATA boxes and polyadenylation signals are indicated within boxes. The start codon and the two stop codons are underlined and presented in bold font. The coding domain sequence (CDS) spans from 1 bp to 2370 bp, with both the start codon and end codon emphasized in bold text. Interval sequences between 501 bp and 550 bp are repeatedly displayed and marked with a long box. A single base alternation of C/T at position 514 bp of CDS differentiates *B1x7* and *B1x7null*, which is highlighted with a pink background in the text. The forward and reverse primers utilized for cloning the *B1x* gene are highlighted with a gray background in the text. The forward and reverse primers of cleaved amplified polymorphic sequence (CAPS) marker are distinguished by a yellow background. The forward and reverse primers utilized for FQ-PCR are highlighted with a green background in the text.

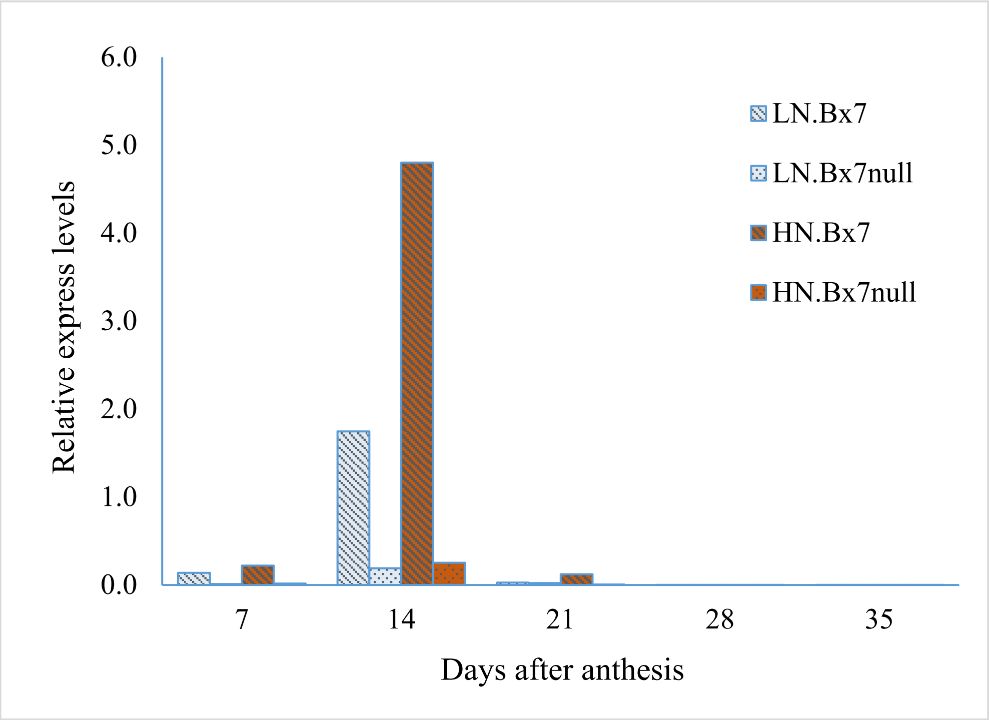

Figure S2. Relative express levels of *Glu-B1x* in Ningmai 9 WT and its NIL under varying nitrogen inputs during the kernel filling stage (Note: LN, low nitrogen input; HN, high nitrogen input)
